# Hearing loss in children with Fabry disease

**DOI:** 10.1007/s10545-017-0051-5

**Published:** 2017-05-31

**Authors:** E. Suntjens, W. A. Dreschler, J. Hess-Erga, R. Skrunes, F. A. Wijburg, G. E. Linthorst, C. Tøndel, M. Biegstraaten

**Affiliations:** 1 Department of Endocrinology and Metabolism and Amsterdam Lysosome Center ‘Sphinx’, Academic Medical Center, University of Amsterdam, Amsterdam, The Netherlands; 2 Department of Clinical & Experimental Audiology, Academic Medical Center, University of Amsterdam, Amsterdam, the Netherlands; 3 Department of Ear, Nose and Throat, Head and Neck, Haukeland University Hospital, Bergen, Norway; 4 Department of Medicine, Haukeland University Hospital, Bergen, Norway; 5 Institute of Clinical Medicine, University of Bergen, Bergen, Norway; 6 Department of Pediatrics and Amsterdam Lysosome Centre ‘Sphinx’, Academic Medical Center, University of Amsterdam, Amsterdam, the Netherlands; 7 Department of Pediatrics, Haukeland University Hospital, Bergen, Norway; 8 Academic Medical Center, Meibergdreef 9, 1105 AZ Amsterdam, The Netherlands

## Abstract

**Background:**

Hearing loss (HL) is a well-known feature of Fabry disease (FD). Its presence and characteristics have mainly been studied in adult patients, while only limited data are available on the presence and degree of HL in children with FD. This prompted us to study hearing sensitivity in pediatric FD patients.

**Methods:**

All available audiograms of the Dutch and Norwegian children with FD were retrospectively collected. First, hearing sensitivity was determined by studying hearing thresholds at low, high, and ultra-high frequencies in children with FD and comparing them to zero dB HL, i.e., healthy children. In addition, the presence and type of slight/mild HL (defined as hearing thresholds at low frequencies of 25–40 dB HL) and moderate to severe HL (hearing thresholds >40 dB HL) at first visit were analyzed. If available, follow-up data were used to estimate the natural course of hearing sensitivity and HL in children with FD.

**Results:**

One-hundred-thirteen audiograms of 47 children with FD (20 boys, median age at first audiogram 12.0 (range 5.1–18.0) years) were analyzed. At baseline, slight/mild or moderate to severe HL was present in three children (6.4%, 2 boys). Follow-up measurements showed that three additional children developed HL before the age of 18. Of these six children, five had sensorineural HL, most likely caused by FD. Compared to healthy children (zero dB HL), FD children showed increased hearing thresholds at all frequencies (*p* < 0.01), which was most prominent at ultra-high frequencies (>8 kHz). Hearing sensitivity at these ultra-high frequencies deteriorated in a period of 5 years of follow-up.

**Conclusion:**

A minority of children with FD show slight/mild or moderate to severe HL, but their hearing thresholds are poorer than the reference values for normal-hearing children. Clinical trials in FD children should demonstrate whether HL can be prevented or reversed by early treatment and should specifically study ultra-high frequencies.

**Electronic supplementary material:**

The online version of this article (doi:10.1007/s10545-017-0051-5) contains supplementary material, which is available to authorized users.

## Introduction

Fabry disease (FD) is an X-linked lysosomal storage disease, characterized by deficient activity of the lysosomal enzyme alpha-galactosidase A (AGAL), which leads to globotriaosylceramide (Gb3) depositions in the lysosomes of various cell types and increased plasma globotriaosylsphingosine (lysoGb3) levels (Brady et al 1967, Aerts et al 2008). Known features of FD are stroke/TIA, cardiac complications and kidney failure (Mehta et al 2009; Germain et al 2002; Rombach et al 2010). The disease is categorized into the severe, classical phenotype, most often seen in males with little or no residual enzyme activity, and the non-classical phenotype, characterized by a more variable disease course. The availability of enzyme replacement therapy (ERT) in 2001 (Eng et al 2001; Schiffmann et al 2001) brought improvement in clinical management of FD patients.

Sensorineural hearing loss (HL) is also a well-known feature of FD (Germain et al 2002). In a recent paper we studied hearing sensitivity in adult FD patients and confirmed that hearing is more severely impaired in male patients with classical FD as compared to male patients with the non-classical phenotype (late onset variants) and female patients. We also showed that in this adult cohort hearing impairment was already present at baseline, and was not reversed by treatment with ERT (Suntjens et al 2015). It was therefore concluded that deterioration of hearing must have started earlier in life. However, evidence on the presence of impaired hearing in children with FD is limited. While children often report hearing complaints, few show actual (clinical) HL by audiometry (Keilmann et al 2009; Limberger et al 2007). One of these studies, which was based on a post-marketing surveillance database, suggested that high-frequency HL occurs during the second decade of life (Keilmann et al 2009). Detailed studies in a well-defined cohort, however, are lacking.

To gain an insight into the frequency, severity, and course of hearing impairment in children, we retrospectively studied the audiometric evaluations of Dutch and Norwegian children with classical FD. In previous papers, we hypothesized that increased plasma lysoGb3 concentrations may play a role in the pathophysiology of both neuropathic pain and HL (Suntjens et al 2015; Biegstraaten et al 2012). We therefore also studied whether neuropathic pain and hearing impairment were associated in this pediatric FD cohort.

## Methods

### Patients

The Academic Medical Center (AMC) in Amsterdam and the Haukeland University Hospital in Bergen are referral centers for FD in the Netherlands and Norway, respectively. All available audiograms of children with classical FD were retrieved. Classical FD was defined as severely reduced AGAL enzyme activity in leukocytes (< 5% of the mean reference value, males only) and a GLA mutation, and either one or more of the following criteria: i) an increase in plasma (lyso)Gb3 (in the range of classical FD males), ii) ≥1 characteristic features of FD (cornea verticillata, angiokeratoma, neuropathic pain), iii) a family member with classical FD (adapted from Van der Tol et al 2014).

The Fabry disease severity scoring system (DS3) was used to determine disease severity at baseline. Because of the retrospective design of this study, we could not incorporate the patient reported outcome (4/80 points in the scoring system), and therefore used the ‘clinical DS3-score’ as described by Gianni et al (2010). Also, we argued that a glomerular filtration rate (GFR)-slope of zero could be used for all patients, since significant loss of GFR in the pediatric age is not to be expected. For individual scores we refer to Appendix 1. The presence of neuropathic pain was defined as a score > 0 on the acroparesthesia item in the DS3.

### Auditory testing

All baseline and follow-up audiograms of children until the age of 18 years were retrieved until December 2014. Baseline audiometry is defined as the first available audiogram of a specific patient. In all cases, this was the audiogram at the time the patient was diagnosed or suspected to suffer from Fabry disease. Audiograms that were performed while the child was treated with ERT were excluded. Also, audiological data of patients who suffered from otological disease at the time of audiometry (e.g., otitis media) as described in their medical history or on the audiogram result were excluded. Follow-up time was defined as the number of years between first and last available audiogram, while untreated.

Audiological evaluation consisted of pure tone audiometry: air conduction thresholds for the conventional range of frequencies (0.25, 0.5, 1, 2, 4, and 8 kHz) and ultra-high frequencies (10, 12, 14, and 16 kHz), and bone conduction (0.5–8 kHz) if air condition was abnormal. Frequencies above 8 kHz were not routinely measured in Norway and are therefore missing in the Norwegian patients (*n* = 19).

Hearing sensitivity was assessed by the following hearing thresholds:

- Average air conduction thresholds at low frequencies (0.5, 1, and 2 kHz): pure-tone average (PTA)_.5,1,2_
- Average air conduction thresholds at high frequencies (4 and 8 kHz): PTA_4,8_
- Average air conduction thresholds at ultra-high frequencies (10–16 kHz): PTA_10,12,14,16_

For the low and high frequencies hearing thresholds are expressed as dB HL. However, for the ultra-high frequencies normal reference values are not available, and it is common use to express the thresholds at frequencies >8 kHz as dB SPL (sound pressure level).

Healthy children not suffering from any otological disease are assumed to have thresholds of zero dB HL.

### Definition of slight/mild and moderate to severe HL

In line with the WHO criteria we defined HL on the basis of low frequency thresholds (PTA_.5,1,2_) of the most severely affected ear, irrespective of type of HL, with a PTA_.5,1,2_ of 25–40 dB HL as cut-off value for the presence of slight/mild HL. Patients suffering from hearing thresholds >40 dB HL were considered to have moderate to severe HL, as they are usually regarded as candidates for wearing hearing aids (International classification of impairments, disabilities, and handicaps WHO May 1980, Geneva, Switzerland).

Asymmetric HL was defined as an inter-aural difference of more than 10 dB for PTA_.5,1,2_ between right and left ear.

### Type of HL

In patients with slight/mild (PTA_.5,1,2_ 25–40 dB HL) or moderate to severe (PTA_.5,1,2_ > 40 dB HL) HL, the type of HL was classified as sensorineural, conductive, or mixed. Sensorineural HL was diagnosed if the average air-bone gap was ≤10 dB for PTA_.5,1,2_. Conductive HL was defined as an average air-bone gap of >10 dB for PTA_.5,1,2_ and normal bone conduction thresholds (≤25 dB HL). Mixed HL was defined as a bone conduction threshold >25 dB HL in combination with an average air-bone gap of >10 dB.

### Statistical analyses

Statistical analyses were performed using SPSS 20.0 (IBM, Chicago). Baseline results are shown by median and range. Children who developed slight/mild or moderate to severe HL are presented in absolute number and percentage. Mann-Whitney U and Chi-square tests were used to compare groups. To determine whether the hearing thresholds in children with FD differ from healthy children (zero dB HL), the Wilcoxon signed-rank test was used, with PTA data as continuous variable and zero as hypothesized median. Associations between variables are described with the use of Spearman’s rho. To study whether hearing thresholds deteriorated over time, we compared baseline data with data at 2 and 5 years follow-up with the Wilcoxon matched pairs signed rank sum test. A *p*-value <0.05 was considered statistically significant.

### Ethical approval

According to Dutch law this retrospective study does not require approval by the Ethical Committee. Patients consented to use their anonymized data as collected in the AMC database for research purposes. The study was approved by the regional ethics committee of Western Norway.

## Results

### Patients

Baseline audiograms were available for 28 Dutch and 19 Norwegian children with classical FD. In addition, 66 follow-up audiograms were included. Median age at time of first audiogram was 12.0 years (range 5.1–18.0 years) (see Table 1). There was no significant difference in age between boys and girls (*p* = 0.24). Boys were more severely affected, demonstrated by higher DS3 scores at baseline (7 vs 3, *p* = 0.002).

**Table 1:** Baseline characteristics

	Boys	Girls	*p*-value
n	20	27	
Age (years); median *(range)*	11.6 *(5.1–17.9)*	12.1 *(6.2–18.0)*	0.24
DS3 score; median *(range)*	7 *(1–15)*	3 *(0–12)*	0.002
Follow up (years); median *(range)*	2 *(0–11)*	2 *(0–8)*	
Baseline (sub)clinical HL; n *(%)*	2 *(10.5%)*	1 *(3.6%)*	

Hearing loss was defined on the basis of low frequency thresholds (PTA_.5,1,2_) of the most severely affected ear. HL = hearing loss, DS3 = disease severity scoring system

### The prevalence of slight/mild and moderate to severe HL at baseline

At baseline, slight/mild HL (PTA_.5,1,2_ 25–40 dB HL) was present in two out of 47 children (4.3%, one boy) while one boy (2.1%) had moderate to severe HL (PTA_.5,1,2_ > 40 dB HL). Two of these three children (both boys) had sensorineural HL, see also Table 2a. Both boys were subsequently treated with ERT in the context of the Fabrazyme: Intervening Early at Low Dose (FIELD) study (Wijburg et al 2015), and therefore no follow-up measurements (while untreated) were available. Also, follow-up audiological data of the 13-year girl were not available. HL was bilateral in one patient (conductive in the left ear, sensorineural in the right) and unilateral in two patients (*n* = 1 sensorineural and *n* = 1 conductive HL). In one patient, HL was asymmetric (26.6 dB inter-aural difference). For baseline audiogram results of each individual patient we refer to Appendix 2.

**Table 2:** a: (Sub)clinical hearing loss at baseline. b: Incidence of (sub)clinical hearing loss

a						
Gender	Age	DS3	Type of HL	PTA_.5,1,2_ (dB HL)	PTA_4,8_ (dB HL)	PTA_10,12,14,16_ (dB SPL)
Boy	5	6	Sensorineural (right ear); Conductive (left ear)	33.3	40.0	Not available
Boy	9	4	Sensorineural	43.3	42.5	Not available
Girl	13	6	Conductive	30.0	32.5	61.7
b						
Gender	Age at onset of HL	DS3	Type of HL	PTA_.5,1,2_ (dB HL)	PTA_4,8_ (dB HL)	PTA_10,12,14,16_ (dB SPL)
Boy	11	12	Sensorineural (two audiograms showed mixed HL)	63.3	52.5	Not available
Girl	15	6	Sensorineural	26.7	30.0	56.3
Girl	12	11	Mixed	28.3	25.0	55.0

Hearing loss was defined on the basis of low frequency thresholds (PTA_.5,1,2_) of the most severely affected ear. HL = hearing loss, DS3 = disease severity scoring system

### Hearing thresholds at baseline

The children in our cohort had a median PTA_.5,1,2_ of 6.7 dB HL (range − 1.7-43.3 dB HL) and a median PTA_4,8_ of 10.0 dB HL (range − 2.5-42.5 dB HL). The median hearing threshold at ultra-high frequencies (>8 kHz, Dutch patients only) was 41.6 dB SPL (range 15–84 dB SPL). These hearing thresholds were worse for the children with FD than the reference values for normal-hearing children (*p* < 0.01) for all frequencies. Boys did not show higher hearing thresholds than girls at low, high, and ultra-high frequencies (*p* = 0.06, *p* = 0.06, and *p* = 0.09, respectively).

### Disease severity

Hearing sensitivity did not correlate significantly with disease severity (DS3 score) for low (*r* = 0.58, *p* = 0.05), high (*r* = 0.13, *p* = 0.39) or ultra-high frequencies (*r* = 0.04, *p* = 0.9).

### Follow-up measurements

**Fig. 1:**
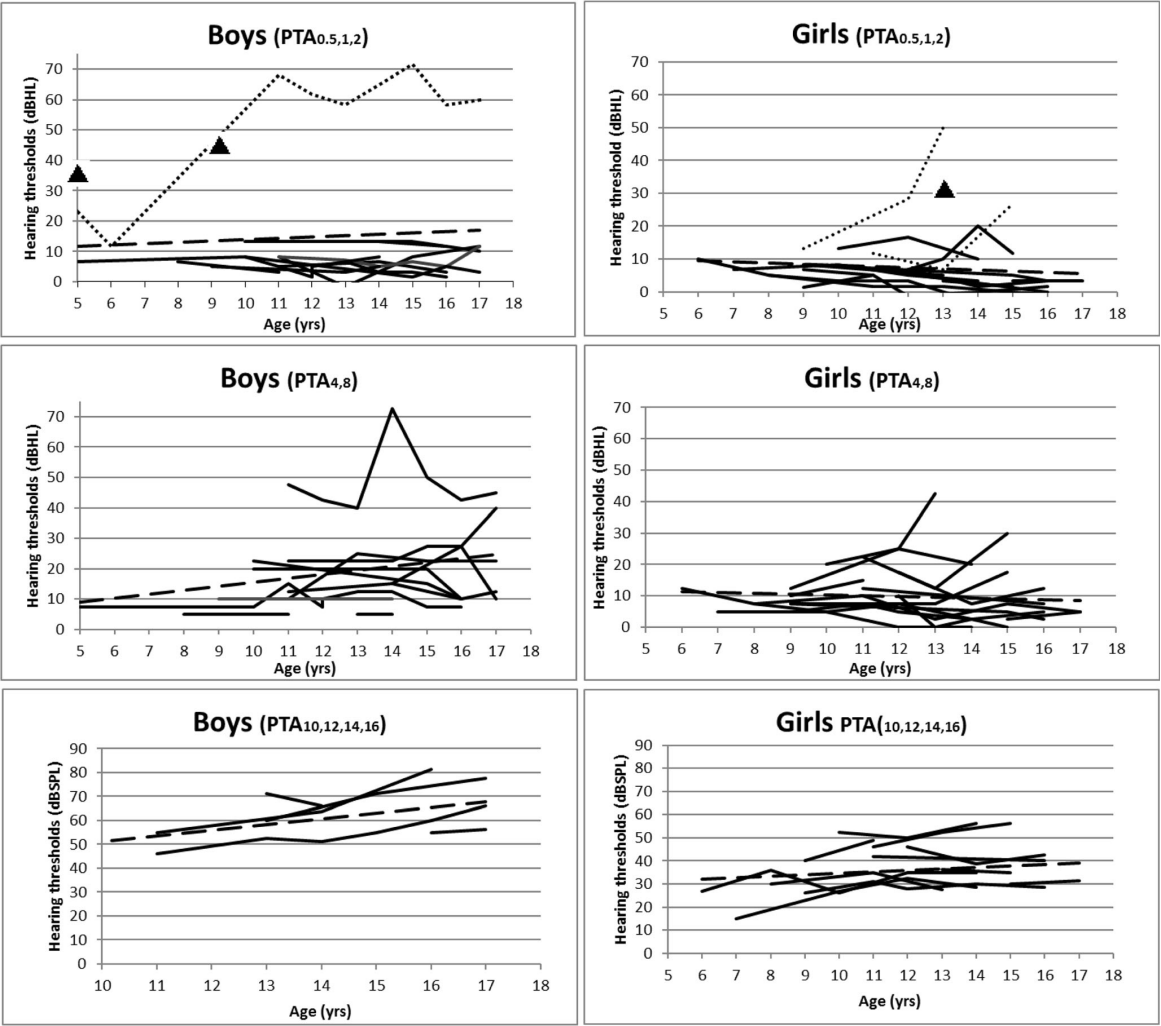
Individual audiometric results of all follow-up data for low (PTA_.5,1,2_), high (PTA_4,8_), and ultra-high (PTA_10,12,14,16_) frequencies. Boys are shown in the left column, girls in the right. Dashed line is the trend-line. Spotted line in PTA_.5,1,2_ demonstrates the three children who developed HL during follow-up. Triangles represent children with HL of whom only one audiogram was available

Follow-up data were available for 26 children, of whom 16 had follow-up data at ultra-high frequencies. Median follow-up was 2 (range 1–11) years. Three children (two girls) did not have HL at baseline, but developed slight/mild HL (PTA_.5,1,2_ 25–40 dB HL) during follow-up. HL in these children was persistent during follow-up. Of these three children, two developed moderate to severe sensorineural HL (>40 dB HL) under the age of 18 years, see also Table 2b and Fig. 1.

Follow-up data after two years were available for 16 children. Hearing thresholds at first audiogram did not differ from thresholds at *t* = 2 years at the low (*p* = 0.67), high (*p* = 0.19) or ultra-high frequencies (*p* = 0.10). Thirteen patients had additional follow-up data at *t* = 5 years. Again, no differences were found at the low (*p* = 0.14) or high frequencies (*p* = 0.83), but the hearing thresholds at the ultra-high frequencies (>8 kHz) had increased (*p* = 0.03). It can be derived from Fig. 1 that HL at low frequencies is rare in the pediatric age (*n* = 6). Hearing thresholds at high and ultra-high frequencies seem to increase, although proper interpretation of these results is hampered by the lack of reference values.

As can be derived from Fig. 1, hearing thresholds of one of the included patients suddenly increased at both low and high frequencies. Criteria for sensorineural HL were fulfilled during 10 out of 12 examinations, while two examinations showed mixed HL. This 11-year old Norwegian boy did not have overt middle ear infection. At the time he experienced sudden deafness, there were no other FD related events, and his DS3 score did not worsen. Since sudden deafness is unusual in children with FD, other causes were sought for. The family history disclosed that his father who does not have FD, experienced sudden deafness of unknown etiology during childhood. It was therefore unclear if the boy’s sudden deafness could be fully explained by FD.

### Hearing thresholds in relation to neuropathic pain

The majority of children (*n* = 34, 72.3%, of whom 18 (53%) boys) suffered from neuropathic pain. These children had worse hearing sensitivity than children without pain at both low (median 14 dB HL vs 6 dB HL, *p* = 0.02) and high frequencies (median 17 dB HL vs 9 dB HL, *p* = 0.04). At ultra-high frequencies there was no significant difference between these two groups (median 46 dB SPL vs 40 dB SPL, *p* = 0.52). Children who had slight/mild or moderate to severe HL (defined as PTA_.5,1,2_ 25–40 dB HL and >40 dB HL, respectively) did not report neuropathic pain more often compared to children without (sub)clinical HL (*p* = 0.46).

## Discussion

In this retrospective study, we evaluated hearing sensitivity of Dutch and Norwegian children with classical FD. Both boys and girls with FD showed increased hearing thresholds compared to the reference values for normal-hearing children at all frequencies. The differences were most prominent for the ultra-high frequencies. Using the stringent criteria for slight/mild and moderate to severe HL (PTA_.5,1,2_ 25–40 and >40 dB HL, respectively), only three children (6.4%) had slight/mild HL at baseline, and an additional three children (6.4%) developed slight/mild HL before the age of 18, of whom five (10.6%) fulfilled the criteria for sensorineural HL. These results are in agreement with our hypothesis that HL may develop in childhood (Suntjens et al 2015). So far, studies on HL in FD children were mainly based on data from post-marketing surveillance databases (Keilmann et al 2009), except for one small retrospective study in the Japanese FD cohort (Sakurai et al 2009). Follow-up data are mostly lacking in these studies and the quality of the dataset used in these studies is often not clear, which may result in over- or underreporting (Hollak et al 2011). Therefore, it is important to note that the reported incidence of HL in 16–19% of FD patients in these studies (Keilmann et al 2009; Hegemann et al 2006) is substantiated with the 10.6% of children who developed sensorineural HL before the age of 18 in this study.

The pathophysiological mechanism leading to increased hearing thresholds and eventually HL in FD is unclear. In an earlier study we found an association between lysoGb3 exposure and small nerve fiber neuropathy which is assumed to underlie the neuropathic pain that is characteristic of FD. Since small fiber neuropathy and HL are both diseases of the nervous system, we questioned whether neuropathic pain and hearing impairment shared the same pathophysiological mechanism and thus were linked. Indeed, children with neuropathic pain showed higher hearing thresholds compared to children without pain. This is in line with a previous study that revealed that HL was more severe in males with small fiber neuropathy (Ries et al 2007). Future studies in which neuropathic pain characteristics, hearing sensitivity, and plasma lysoGb3 concentrations are measured simultaneously may support the hypothesis of a common pathophysiological pathway.

Interestingly, hearing problems in children have been described in several other lysosomal storage disorders (LSDs) as well, including type 3 Gaucher disease (Bamiou et al 2001), the mucopolysaccharidoses (Lin et al 2014; Keilmann et al 2012), and Pompe disease (van Capelle et al 2010). In these diseases, both mixed and sensorineural HL has been reported. Despite the fundamental differences in accumulated substrate in each of these diseases, the cochlea, auditory nerve or brainstem is invariably affected which in turn results in HL. In our study we demonstrated that in FD, hearing thresholds at ultra-high frequencies (>8 kHz) are more severely increased than thresholds at lower frequencies. Increased thresholds at low and high frequencies with a clinically significant decline in hearing sensitivity from baseline in a subset of patients have been reported in patients with Niemann-Pick disease type C (King et al 2014). It is surprising that, besides this study, only one other study has been performed on the natural course of HL in children with LSDs. This study of MPS patients showed that age was a major determinant of slight/mild or moderate to severe sensorineural HL, which started at the age of 11–15 years, especially in MPS type II (Lin et al 2014). The effect of ERT on HL prevention or slowing of progression has not been well studied to date. In Pompe disease, hearing sensitivity assessed by brainstem evoked potentials and otoacoustic emissions, but not by audiometry, demonstrated that hearing thresholds in 10 patients did not change after a period up to 6 years of ERT treatment (van Capelle et al 2010). This is in line with the observations we made in adult Fabry patients (Suntjens et al 2015). Altogether, our study highlights the importance of measuring ultra-high frequencies (>8 kHz) when evaluating both the natural course of hearing sensitivity and the possible impact of ERT for all LSDs, especially since it appears to be the first marker to show abnormalities.

Despite the fact that we studied a well-defined cohort, our study has some limitations; we did not evaluate a potential effect of ERT in our patient group, as most ERT treated children participated in the FIELD study (ClinicalTrials.gov NCT00701415) and its results have not yet been published. The remaining data on ERT treated pediatric patients (not participating in that study) were too limited and therefore excluded. The exclusion of follow-up of patients treated with ERT in the FIELD study may subsequently have led to a bias in this study, because it excludes the most severely affected patients (those who needed treatment with ERT). When the outcome of the FIELD study is published, new light may be shed on the effectiveness of ERT on HL in children. One other challenge in studying hearing sensitivity in children is the relatively high incidence of middle ear problems (Todberg et al 2014). We excluded audiograms of children who were reported to have otitis media, but due to the retrospective design of our study we had to rely on what has been written in the medical charts which may have led to underestimation of the presence of otitis media. Also, slight/mild otitis may have gone unnoticed which could have influenced our results. In particular, the finding of conductive HL in one out of the six children with HL is probably best explained by unrecorded or unnoticed middle ear infection. If only sensorineural HL is considered to be caused by FD, we found five children (10.6%) with HL due to FD under the age of 18.

In summary, impaired hearing sensitivity is common in children with FD, although only a few children fulfill the audiometric criteria of slight/mild HL (4.3%) or moderate to severe sensorineural HL (6.4%). Since hearing thresholds at the ultra-high frequencies are particularly increased, we recommend to include these frequencies in follow-up audiograms. It remains to be evaluated whether increased thresholds or sensorineural HL should be considered an indication to start ERT.

## Electronic supplementary material

ESM 1 (DOCX 23.5 kb)

ESM 2 (DOCX 15.7 kb)
