# Supplementary material for: Hearing loss in children with Fabry disease
Source: J Inherit Metab Dis. 2017 May 31;40(5):725–31. doi: 10.1007/s10545-017-0051-5 (PMC5579138; doi:10.1007/s10545-017-0051-5)
Supplement: Supplementary file 2 — (DOCX 15.7 kb) [file 10545_2017_51_MOESM2_ESM.docx]

**Appendix 2. Individual baseline audiogram results**

| **Pt** | **Gender (M/F)** | **Age** | **DS3** | **PTA_.5,1,2_ (dB HL)** | **PTA_4,8_ (dB HL)** | **PTA_10,12,14,16_ (dB SPL)** |
| --- | --- | --- | --- | --- | --- | --- |
| 1 | M | 11 | 1 | 5.0 | 12.5 | 55.0 |
| 2 | M | 11 | 8 | 5.0 | 5.0 | 27.0 |
| 3 | M | 12 | 11 | 5.0 | 2.5 | 30.0 |
| 4 | M | 11 | 9 | 8.3 | 10.0 |  |
| 5 | M | 5 | 6 | 33.3 |  |  |
| 6 | M | 14 | 13 | 13.3 | 15.0 |  |
| 7 | M | 17 | 15 | 8.3 | 22.5 | 61.25 |
| 8 | M | 11 | 13 | 8.3 | 22.5 | 46.25 |
| 9 | M | 9 | 4 | 43.3 | 42.5 |  |
| 10 | M | 5 | 11 | 6.7 | 7.5 |  |
| 11 | M | 5 | 12 | 23.3 |  |  |
| 12 | M | 8 | 1 | 6.7 | 15.0 |  |
| 13 | M | 10 | 6 | 8.3 | 20.0 |  |
| 14 | M | 14 | 13 | 5.0 | 5.0 |  |
| 15 | M | 13 | 12 | 5.0 | 5.0 |  |
| 16 | M | 14 | 6 | 10.0 | 25.0 |  |
| 17 | M | 10 | 6 | 13.3 | 22.5 |  |
| 18 | M | 9 | 5 | 5.0 | 10.0 |  |
| 19 | M | 13 | 5 | 6.7 | 12.5 | 71.25 |
| 20 | F | 12 | 6 | 20.0 | 15.0 | 84.0 |
| 21 | F | 12 | 1 | 6.7 | 17.5 | 46.25 |
| 22 | F | 11 | 6 | 11.7 | 22.5 | 46.25 |
| 23 | F | 17 | 0 | 3.3 | -2.5 | 34.0 |
| 24 | F | 10 | 1 | 13.3 | 20.0 | 52.5 |
| 25 | F | 13 | 3 | 3.3 | 7.5 | 36.25 |
| 26 | F | 9 | 10 | 1.3 | 2.5 | 25.0 |
| 27 | F | 11 | 6 | 6.7 | 12.5 | 42.0 |
| 28 | F | 10 | 1 | -1.7 | 7.5 | 57.5 |
| 29 | F | 15 | 0 | 1.7 | 10.0 |  |
| 30 | F | 12 | 10 | 6.7 | 10.0 |  |
| 31 | F | 9 | 11 | 13.3 | 12.5 |  |
| 32 | F | 17 | 0 | 1.7 | 10.0 |  |
| 33 | F | 12 | 1 | 10.0 | 15 |  |
| 34 | F | 13 | 5 | 8.3 | 0.0 |  |
| 35 | F | 9 | 0 | 8.3 | 7.5 |  |
| 36 | F | 16 | 0 | 5.0 | 0.0 |  |
| 37 | F | 14 | 0 | 5.0 | 7.5 |  |
| 38 | F | 9 | 11 | 8.3 | 7.5 |  |
| 39 | F | 17 | 6 | 6.7 | 7.5 | 38.75 |
| 40 | F | 17 | 12 | 5.0 | 10.0 | 42.5 |
| 41 | F | 8 | 9 | 5.0 | 7.5 | 30.0 |
| 42 | F | 13 | 6 | 30 | 32.5 | 61.7 |
| 43 | F | 6 | 5 | 10 | 12.5 | 27.0 |
| 44 | F | 7 | 1 | 6.7 | 5.0 | 15.0 |
| 45 | F | 16 | 5 | 5.0 | 7.5 | 41.25 |
| 46 | F | 15 | 0 | 3.3 | 2.5 | 30.0 |
| 47 | F | 9 | 0 | 6.7 | 10.0 | 40.0 |
